# DESTINI: A deep-learning approach to contact-driven protein structure prediction

**DOI:** 10.1038/s41598-019-40314-1

**Published:** 2019-03-05

**Authors:** Mu Gao, Hongyi Zhou, Jeffrey Skolnick

**Affiliations:** Center for the Study of Systems Biology, School of Biological Sciences, Georgia Institute of Technology, Atlanta, GA 30332 USA

## Abstract

The amino acid sequence of a protein encodes the blueprint of its native structure. To predict the corresponding structural fold from the protein’s sequence is one of most challenging problems in computational biology. In this work, we introduce DESTINI (deep structural inference for proteins), a novel computational approach that combines a deep-learning algorithm for protein residue/residue contact prediction with template-based structural modelling. For the first time, the significantly improved predictive ability is demonstrated in the large-scale tertiary structure prediction of over 1,200 single-domain proteins. DESTINI successfully predicts the tertiary structure of four times the number of “hard” targets (those with poor quality templates) that were previously intractable, viz, a “glass-ceiling” for previous template-based approaches, and also improves model quality for “easy” targets (those with good quality templates). The significantly better performance by DESTINI is largely due to the incorporation of better contact prediction into template modelling. To understand why deep-learning accomplishes more accurate contact prediction, systematic clustering reveals that deep-learning predicts coherent, native-like contact patterns compared to co-evolutionary analysis. Taken together, this work presents a promising strategy towards solving the protein structure prediction problem.

## Introduction

The protein folding problem asks the following question: given an amino acid sequence of a protein, can one predict its corresponding 3D structure? Since this question was raised in the 1960s^1^, a tremendous amount of research efforts have been invested towards its solution. Roughly speaking, the proposed solutions to this question can be categorized into two groups: *de novo* structure prediction and comparative modelling, or equivalently template-free and template-based approaches^2–7^.

For any approach, one key factor that dictates the quality of protein structure prediction is the information about protein residue-residue contacts (or protein contacts), which are usually defined by the distance between protein backbone C_α_ atoms or between sidechain C_β_ atoms. If a sufficient number of such protein contacts are known, especially non-local contacts between residues separated by a long sequential distance >20 residues, one can reconstruct the structural model of a protein. An early study estimates that, for single-domain proteins less than 200 amino acids (AAs), one can assemble a structural model within a 5 Å root-mean-square-deviation (RMSD) from the native structure if more than *L*/4 long-range protein contacts are known^8^, where *L* is the length of the protein. Another study estimates that, on average, one contact per twelve residues allows for robust and accurate protein fold modelling^9^. Overall, protein contact information is the most important component in modern structural modelling packages^3,5,10^. In a recent study, using protein contacts inferred from co-evolutionary constraints by direct coupling analysis, structural models for 614 protein families with currently unknown structures have been generated^7^.

Because of its importance in protein structure prediction, many computational methods have been developed to predict protein contacts. A widely adopted class of methods uses structural templates to infer contacts^11,12^. Given an input sequence, these methods typically employ a “threading” algorithm^2,13^ to identify homologs with experimentally determined structures, and subsequently infer protein contacts from the structures of suitable templates. However, homologous templates are not available for many protein families. Moreover, even though such structural templates are found in the PDB library, for about 15% of single-domain proteins, so-called “glass-ceiling” proteins, threading-based approaches fail because they cannot identify the correct template as multiple contact formation schemes are possible with the best answer often associated with an incorrect structure^14^. Clearly, a different strategy for protein contact prediction is required to address these cases. When the structural template is not available, co-evolutionary analysis methods have proven to be useful. The idea was proposed decades ago^15,16^, and first implemented in the structure prediction algorithm MONSSTER^17^; but at the time, its results were seriously limited due to the small library of evolutionarily related protein sequences that were available. Recently, the explosion of genomics data has rejuvenated co-evolutionary analyses for protein contact prediction, examples include EVfold^18^, CCMpred^19^, Gremlin^20^, PSICOV^21^, plmDCA^22^ and others^23^. Moreover, template or sequence information can be combined to train supervised machine learning approaches, e.g., PconsC2^24^, MetaPSICOV^25^, SVMSEQ^11^, CMAPpro^26^, PhyCMAP^27^ and CoinDCA-NN^28^. These can achieve better performance on proteins without many sequences for co-evolutionary analysis, but their performance improvement is limited and not able to deal with those cases when appropriate template information is lacking.

Recently, a breakthrough in supervised machine-learning gave rise to a new generation of so-called “deep-learning” algorithms, which has been demonstrated as a promising technique for image classification, as well as in speech recognition^29^. In a typical deep-learning algorithm, the main architecture consists of many layers of convolutional neural networks (CNNs). Through these convolutional layers, features could be learned and collected at different levels during training and applied for subsequent classification^30^. As shown in the annual ImageNet Large Scale Visual Recognition Challenge organized since 2010^30,31^, applications based on deep-learning have easily beaten traditional “shallow” machine-learning architectures. On the other hand, the question of protein contact prediction could be converted to an image classification problem at the pixel level, which is equivalent to predicting a residue-residue contact map. In principle, deep-learning algorithms for image recognition could be applied to the prediction of a protein’s contact map. However, since the contact map requires dense pixel-level labelling, not a simple classification of the whole image as in image recognition, one needs to apply a segment-based classification algorithm using fully convoluted neural networks (FCNs)^32^. Very recently, such an idea has been introduced by a couple of groups that employed somewhat different designs, but all used fully convolutional networks for predicting the protein contact map^33,34^. As shown in the most recent 12^th^ Critical Assessment of Structure Prediction (CASP) competition^35^, these new methods achieved significant improvement over previous methods based on co-evolutionary analysis or “shallow” machine-learning techniques.

Despite success in contact prediction, however, it is not straight-forward to translate better contact prediction into better protein structure prediction. In previous studies^33,34^, the main focus was on the improvement of contact prediction accuracy, rather than on the improvement of protein structure prediction quality per se. These studies also lacked a comprehensive comparison to state-of-art structural prediction approaches. Indeed, CASP12 assessors noted that turning a better contact prediction into a better structural model remains a main challenge^35^. To address this key issue, in this work, we introduce DESTINI, a deep-learning based computational approach to address the problem of protein structure prediction. We have performed extensive benchmark tests on over 1,200 protein targets to demonstrate a significant breakthrough in both hard, “glass-ceiling”, targets where template-based methods have a high failure rate^14^, and in easy targets where our approach can further improve the success rate relative to the good models generated by the previously established, state-of-art structural modeling method TASSER^5^. Finally, we also show that our deep-learning contact prediction model achieves better performance compared to existing representative approaches.

## Results

**Figure 1:**
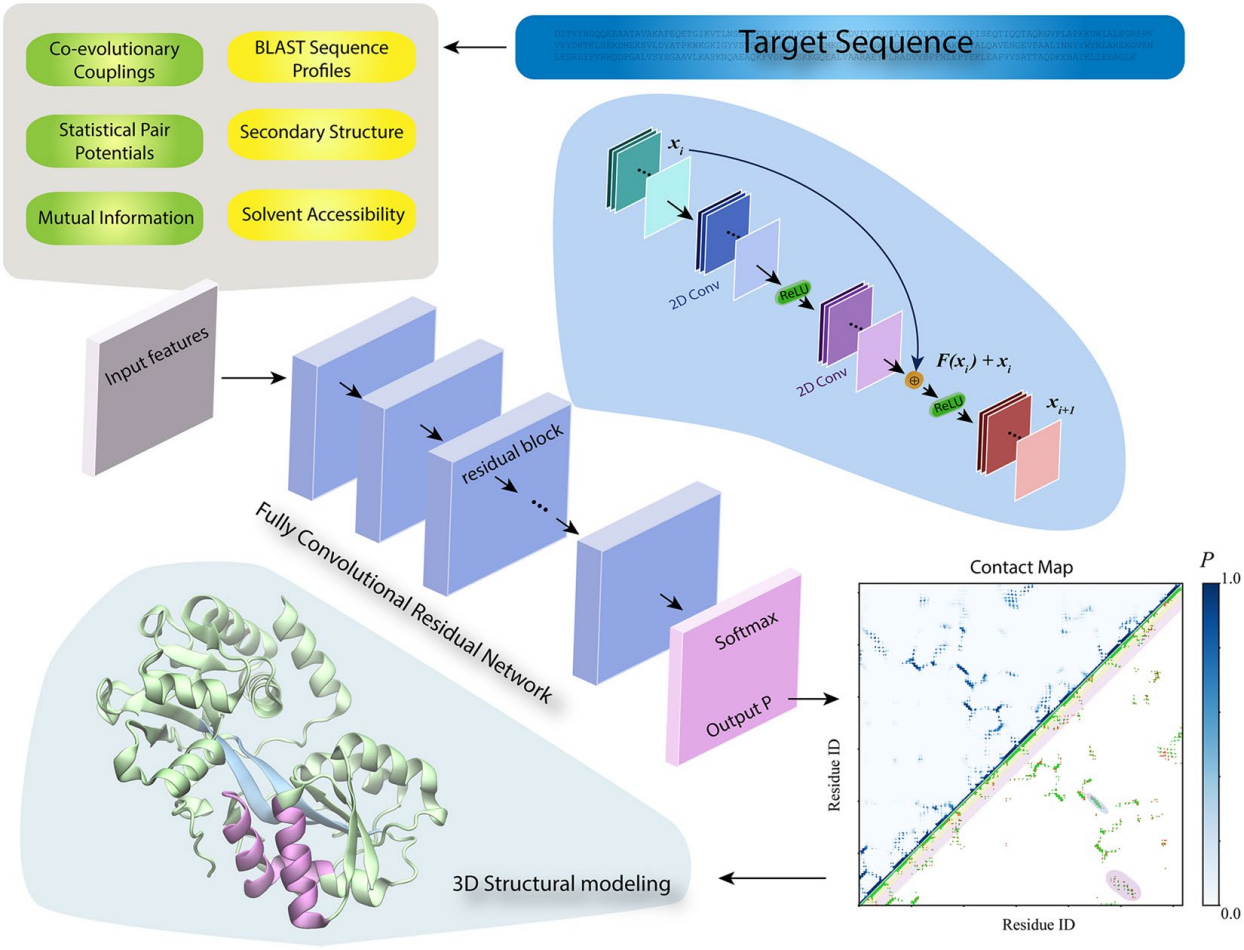
Overview of DESTINI. Given an input target protein sequence, 1D (yellow rectangles) and 2D (green rectangles) features are extracted by mining existing genomic and structural data. These features are inputs to a fully convolutional residual network, a deep-learning artificial neural network composed of multiple identical residual blocks, whose architecture is shown in the blue bubble. The final layer of the network is a softmax activation layer, which outputs the probability score (*P*) for every pair of residues of the target sequence. The output is displayed in a contact map plot, where the upper triangle displays the probability scores colored according to the scale on the right, and the lower triangle plots a comparison of the predicted contacts (*P* > 0.5) versus true (native) contacts. Green, red, grey dots are true positives, false positives, and false negatives, and short/medium range contacts are represented by yellow/pink stripes. The predicted contacts are subsequently employed to derive the 3D model of the target. The final 3D structure of the target is exhibited in a cartoon representation. The purple α-helix/α-helix contacts and cyan β-strand/β-strand contacts correspond to the contacts shown in the contact map shaded using the same color code.

An overview of DESTINI is illustrated in Fig. 1. Given a protein sequence, it predicts an atomic 3D structural model for the target sequence. DESTINI has two main components: contact prediction and structural modeling. The contact prediction is an implementation of a fully convolutional residual neural network composed of 102 layers in total, including 40 convolutional layers (see Methods). The input features consist of three 2D features: co-evolutionary coupling scores^19^, a statistical potential^36^, and mutual information for pairs of residues^37^, and three 1D features: BLAST sequence profiles^38^, secondary structure and solvent accessibility predictions^25^, which are converted into 2D features by concatenating 1D features of two separate residues of a residue pair. The contact predictions are then supplied to structural modeling, the second component of DESTINI, which is a further development based on the TASSER^VMT^ approach (abbreviated as TASSER below)^5^. When there is no suitable template model available, the structural modeling essentially makes *de novo* predictions^4^; if there is a significant structural template hit, modeling based on template(s) is conducted. In both scenarios, confident contact predictions serve as the main driver towards the native structural fold.

Below, we describe the benchmark results on three testing sets. A contact between a pair of protein residues *i* and *j* is defined if the Euclidean distance between the C_β_ atoms of these residues (C_α_ atom is used for Glycine) is less than 8 Å. Following CASP procedures^35^, the precision of the top *L/k* contact predictions, where *L* is the length of the target and *k* = 1, 2, 5, 10, are employed as the main metric for evaluation. We only consider *non-local* contacts, i.e. where the sequential distance of residues *i* and *j*, $|i-j|$, falls into three regimes: *short* [6, 11], *medium* [12, 23], and *long* [24, ∞). For practical reasons, the latter two regimes are most valuable for structural modeling, and therefore, they are the focus of our subsequent evaluation. The quality of structural models is measured by their TM-score^39^ with respect to the native (experimentally determined) structure. A TM-score higher than 0.4 indicates a model that significantly resembles the native topology, or is *native-like*, and a score higher than 0.5 suggests a highly similar structure to the native fold^40^.

### Deep-learning predicts contacts are more accurate than the co-evolutionary or template-based approach

**Figure 2:**
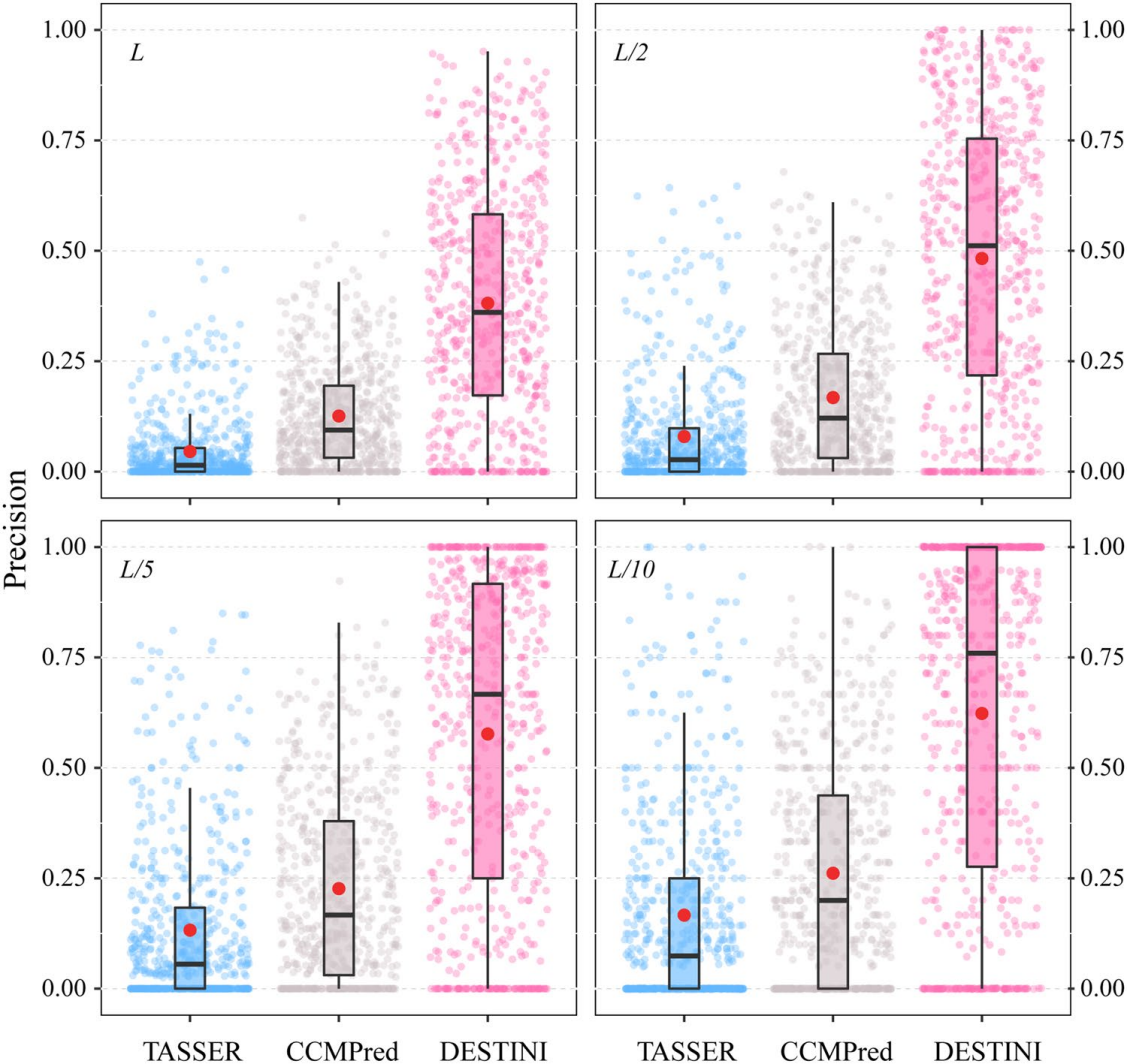
Precision of medium/long range contact predictions on 606 hard targets. Top *L*/*k* (*k* = 1, 2, 5, 10) predictions are shown in the four panels. In each panel, three boxplots display the results of three methods, TASSER (blue), CCMPred (grey), and DESTINI (pink), respectively. In each boxplot, the black box indicates the interquartile range from 25% to 75%; the median is represented by a black bar within the box; and the whisker extends up to 1.5 times the interquartile range. The red circle is positioned at the mean value. Individual data points from each method are shown as small transparent circles in the same color code as the boxplot.

We first evaluate the contact predictions for the “glass-ceiling” set, which is a set of 606 hard targets that are difficult for the classical threading algorithms to identify correct structural templates^14^. In this and the next test on the “easy” set, we excluded any protein in the training set or in the template library if it shares a sequence identity of 30% or higher with any target in the testing set. This procedure is necessary in order to obtain objective evaluations. Figure 2 shows the precision of contact prediction for medium or long-range contacts for three methods, TASSER, CCMPred^19^ and DESTINI. Since this set was created using hard targets for TASSER, as expected, contact prediction by TASSER using a template consensus scheme delivers poor results: the mean precision is merely 4.6% for the top *L* predictions, and 8.0%, 13.3%, and 16.7% for top *L*/2, *L*/5, and *L*/10 predictions, respectively. The co-evolutionary analysis method CCMPred^19^ doubles these accuracies over TASSER for this set, with the corresponding top *L/k* (*k* = 1, 2, 5, 10) predictions at 12.6%, 16.8%, 22.7%, and 26.1%, respectively. Compared to CCMPred, whose scores are the most important input feature for our deep-learning neural network, DESTINI further significantly improves the accuracy of contact prediction. For the top *L* predictions, the mean precision per target is 38.1%, triple the mean precision of CCMPred at 12.6%. Similarly, the mean precision for the top *L*/2, *L*/5, and *L*/10 sets shows dramatic improvement over CCMPred from 16.8%, 22.7%, and 26.1% to 48.3%, 57.3% and 62.3% by DESTINI, respectively. On over half of the targets, DESTINI achieves a precision better than 50% for the top *L/*2 predictions, with median precision values of 67% and 75% for the top *L/*5 and *L/*10 predictions.

Note that there are 46 (7.6%) targets that are short helical proteins without any medium/long range contacts observed in the native structure, e.g., a single helix. For 24 (52%) of them, DESTINI correctly makes no positive contact prediction, i.e., a probability score *P* < 0.5. However, they are still assigned zero precision values in order to be consistent with the CASP evaluation scheme, which selects top predictions even when a score is not significant. This consideration is due to the fact that some methods, such as CCMPred, do not provide a score cutoff for positive, i.e. confident, contact predictions.

### Deep-learning detects and expands contact patterns over the co-evolutionary approach

**Figure 3:**
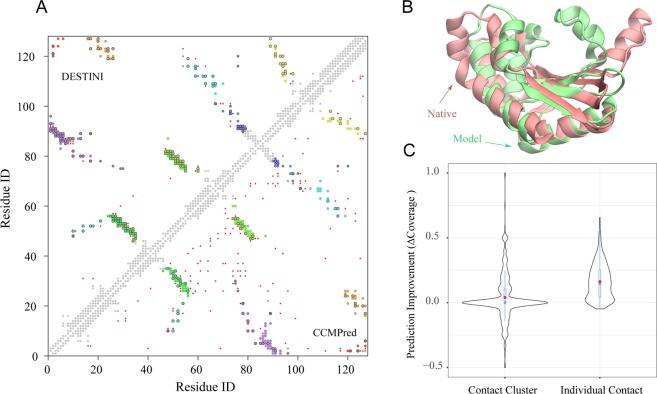
Example and statistical analysis of contact pattern prediction. (**A**) A representative example of the native contact map *versus* the predicted contact map by DESTINI (upper triangle) and by CCMPred (lower triangle). The target is TT1751 of *T. thermophilus* HB8 (PDB code 1J3M)^41^. Medium and long range native contacts are represented by circles filled in different colors for different contact clusters, except that isolated, unclustered contacts are represented by grey circles. Correctly predicted contacts by either method are indicated by black borders surrounding the circles. False positives are represented by red dots. Local and short range native contacts are displayed in light grey squares along the diagonal. (**B**) Superposition of the top structural model by DESTINI (red) onto the native structure (green). Models are shown in cartoon representations using the visualization program VMD^53^. (**C**) Violin plots of ΔCoverage for contact clusters and individual clustered contacts, respectively, for targets of the “glass-ceiling” set. In each violin, the black contour exhibits the probability density estimated using the data set. The blue boxplot inside the violin follows the same boxplot scheme adopted in Fig. 2.

Where does the improvement come from? A representative successful example provides some hint, as shown in Fig. 3A. This is a little known TT1751 of *T. thermophilus* HB8, a 127 AA protein with unknown biological function but nevertheless solved crystal structure^41^. The observed 192 native medium/long range contacts of this target can be largely grouped into 11 geometric clusters, each with at least 3 contacts, and only a few, 12 scattered contacts do not belong to any cluster. These clusters correspond to typical contact patterns between β-strands, α-helixes, and β-strands/α-helixes as seen in the native structure (Fig. 3B). DESTINI predicts a total of 202 contacts (*P* > 0.5) and 152 of them are correct, hitting all 11 clusters and recalling 82.7% of individual contacts in these clusters. By comparison, if one considers the same number of top ranked contact predictions by CCMPred, it also hits all native clusters but with a much smaller coverage of individual contacts within these clusters, only 33.3%. Instead, CCMPred leaves many false positives scattered on the contact map (Fig. 3A). Therefore, this suggests that deep-learning recognizes clusters corresponding to contact patterns and promotes true positives within the clusters, while it also reduces isolated false positives not around the clusters. Even among the 50 false positives by DESTINI, 38 (76%) surround the native clusters with no more than a two-residue shift. Given these improvements, we are able to fold the structure similar to the native fold at TM-score of 0.577 (Fig. 3B). The overall backbone C_α_ RMSD is 5.8 Å, and 87 (69%) residues have an RMSD of 2.7 Å when superimposed on the crystal structure by TM-align^39^. If we use the number of top ranked contact predictions by CCMPred, we obtained a much worse structural model with a TM-score of 0.437, though it already has a roughly native-like topology with an RMSD of 8.9 Å.

Systematic contact pattern analysis through clustering on the “glass-ceiling” set reinforces the insights gained from the example above. We considered 545 targets, which have at least one contact cluster within the medium or long regime. On average, there are 8.9 clusters per target. Among them, DESTINI detects 4.8 clusters on average, whereas CCMPred hits 4.7 clusters per target. In about half of the cases, DESTINI identifies the same set of clusters discovered by co-evolutionary coupling analysis. However, for these clusters, DESTINI finds on average 8.2 contacts, significantly more than the 3.5 contacts provided by CCMPred. We further calculate ΔCoverage ≡ Coverage_DESTINI_ - Coverage_CCMPred_ (the subscript denotes the method employed to predict contacts) for clusters and individual contacts within cluster hits, respectively. These distributions are shown in Fig. 3C. In 253 (46%) cases, the same number of clusters are hit by both approaches; in 173 (32%) of cases DESTINI predicts more clusters, whereas in 119 (22%) cases CCMPred finds more clusters. On average, DESTINI improves cluster coverage by 0.04, which is very small, but statistically highly significant (Wilcoxon pair test *P-value* = 1.8 × 10^−6^). By contrast, ΔCoverage of individual contacts that have a mean value of 0.162, and 461 (85%) targets show improvement when DESTINI versus CCMPred is applied. Moreover, DESTINI’s false positives are mostly located around clusters within a two-residue shift. On average, 71% of them surround the native clusters versus 51% of CCMPred’s false positives are found using the same criteria. Overall, the improved contact predictions by DESTINI stem from better recognition of contact patterns within clusters already hit by CCMPred, while pruning isolated false positives.

### Accurate contact predictions yield native-like folds for hard targets

**Figure 4:**
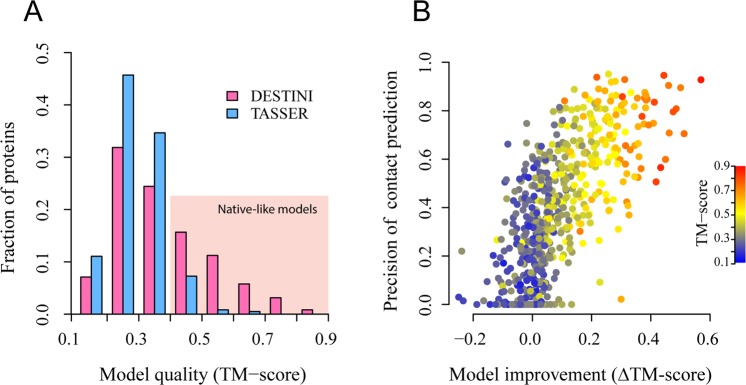
Structural models of DESTINI compared to TASSER models for the “glass-ceiling” set. (**A**) Histograms of TM-scores for each protein target. The shaded area represents good, native-like models. (**B**) Correlation between model quality improvement and the precision of medium/long range contact predictions (top *L*). Each circle represents a target protein.

Using the improved contact predictions, DESTINI’s predicted structural models are significantly better than the models generated by TASSER. Figure 4 demonstrates the comparison of the top models (ranked without using the native structure) by these two approaches. A histogram by the TM-score of the models suggests that much more native-like models are found by DESTINI (Fig. 4A). A total of 222 (36.6%) targets have a top model TM-score > 0.4, compared to only 52 (8.6%) targets under the same criterion by TASSER. The results demonstrate that more than four times the number of native-like models are obtained by the deep-learning based approach. Moreover, among these good models, the quality of the models is generally better by DESTINI, which yields a mean TM-score of 0.539, 18% higher than the mean TM-score of 0.456 by TASSER. If one uses a TM-score 0.5 as the criterion for a good quality prediction, DESTINI folds 127 targets (21.0%) versus merely 8 targets (1.3%) by TASSER. The fractions of mainly α, β, and α/β structures among those with native-like structures is 45%, 10%, and 45%, respectively, compared to the fractions 52%, 12%, and 36%, respectively, in the full set. Thus, the success rate is independent of secondary structure class. Overall, the results clearly demonstrate a significant advantage of DESTINI in predicting protein structural folds.

Further analysis shows that the improvement of model quality by DESTINI over TASSER is strongly correlated to the quality of the contact predictions. A high Pearson correlation coefficient of 0.70 is obtained between the difference of TM-score (ΔTM-score ≡ TM-score_DESTINI_ - TM-score_TASSER_, where the subscript denotes the method employed to predict structural model) and the precision of the top *L* medium or long-range contact predictions. Among 215 targets, which have big, positive model improvement at a ΔTM-score > 0.1, they have a mean contact precision of 61.2%. In contrast, for 167 targets which show no improvement (ΔTM-score ≤ 0), the mean precision for contact prediction is merely 18.0%. The stark contrast suggests, not surprisingly, that better contact prediction is essential to the model improvement by DESTINI.

**Figure 5:**
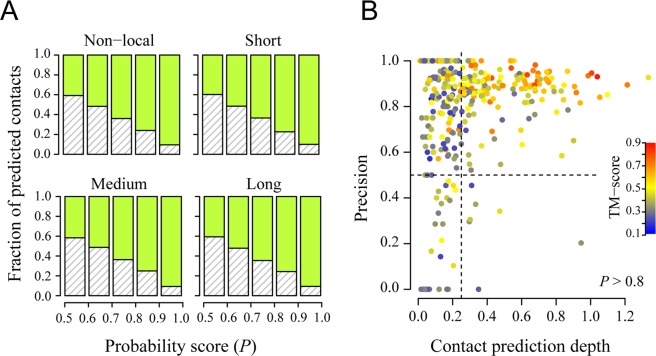
Precision of contact prediction and its impact on structural modeling for the glass-ceiling set. (**A**) Distributions of positive contact predictions at different probabilities for all non-local, short, medium, and long range residue pairs. Green indicates correct predictions and grey hash represents incorrect predictions. (**B**) Precision of predicted medium or long range contacts with a probability score better than 0.8 versus contact prediction depth. Each target is presented by a circle, filled by color corresponding to the TM-score of the model. Insert is the color scale. The same color code is adopted in the subsequent figures. The vertical/horizontal dashed lines are at 0.25 and 0.5, respectively.

One practical question is: how many correct contact predictions are required in order to fold a target protein correctly? Strictly speaking, the answer to this question is dictated by the specific protein fold, and thus, there is no general answer. Nevertheless, for single-domain proteins, as in this glass-ceiling set, one can get an approximate answer to this question. The probability score *P* for contact prediction suggests the confidence level of a prediction. Figure 5A shows that, for all non-local contact predictions, 90.5%/76.0% of positive predictions corresponding to *P* > 0.9/0.8 are correct. The numbers are roughly the same if one separately calculates the precision for the three regimes: 90.1%/77.3% in short, 90.7%/75.1% in medium, and 90.6%/75.7% in long range predictions, corresponding to *P* > 0.9/0.8. If we consider only high quality predictions at *P* > 0.8 for medium or long range contacts, a total of 346 (57%) targets have at least one such prediction. We further define *contact prediction depth D* as the number of considered predictions divided by *L*, the length of a target. As shown in Fig. 5B, the higher the contact prediction depth, the more accurate the resulting structural models. At *D* > 0.2, 0.25, 0.5, the factions of models with a TM-score > 0.4 are 69%, 75%, 84%, respectively. Therefore, it appears that high confidence contact prediction at *D* > 0.2 provides a good chance of obtaining a native-like fold in this single-domain set; this is rather consistent with early results which suggested that *L*/4 such contacts are sufficient^8^.

### Accurate contact predictions improve model refinement for easy targets

**Figure 6:**
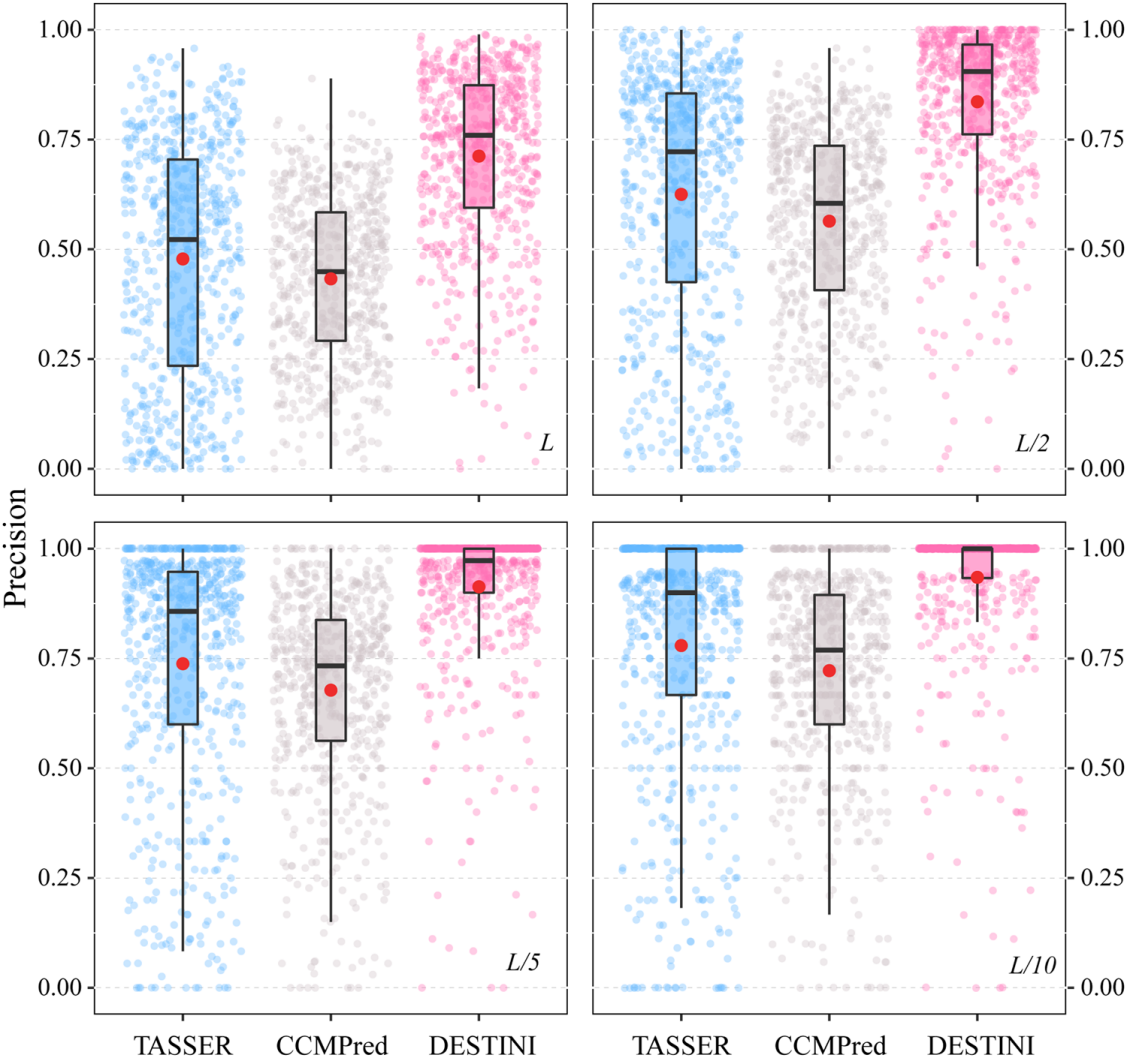
Precision of medium/long range contact predictions on 631 easy targets. The same plot scheme as Fig. 2 is adopted.

Next, we ask the question of whether accurate contact prediction by DESTINI can be utilized to improve the structural models of “easy” protein targets, which already have one or more significant structural templates identified by threading. To address this, we applied DESTINI to 631 targets marked as “easy” by threading approaches^14^. Figure 6 compares the results of contact prediction by DESTINI, TASSER, and CCMPred. On average, these targets have a far better accuracy in contact prediction than the hard targets analyzed above. CCMPred yields a mean precision of 43.3% and 67.8% for the top *L* and *L*/5 ranked scores, respectively, compared to 12.6% and 22.7% of the glass-ceiling set. Template-based predictions by TASSER provides better results: 47.8% and 73.8% for top *L* and *L*/5 predictions on average. Despite the relatively high starting points, our deep-learning approach can further improve the contact prediction by raising the mean precision to 71.2% and 91.3% for the top *L* and *L*/5 ranked scores, respectively. On over 75% of targets, DESTINI yields a high precision over 75% among top *L* scores.

**Figure 7:**
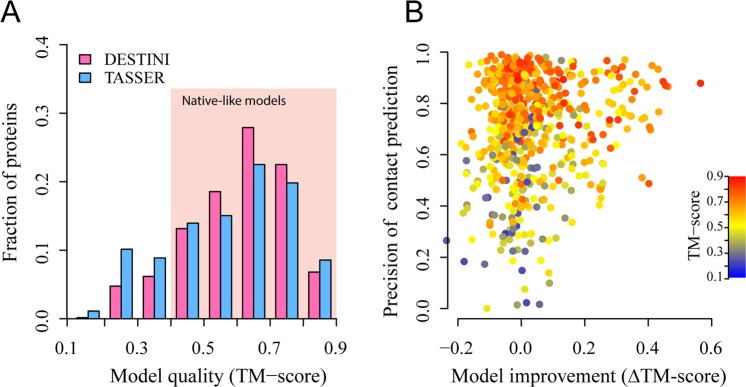
Structural models of DESTINI compared to TASSER models for easy targets. (**A**) Histograms of TM-scores for each protein target. (**B**) Correlation between model quality improvement and the precision of medium/long range contact predictions (top *L*).

Using these accurate contact predictions, DESTINI generates 561 (89%) native-like structural models among the top ranked models for each target versus 504 (80%) native-like models yielded by TASSER (Fig. 7A). This corresponds to an 11% increase in the number of targets with the native-like models. If one uses a TM-score > 0.5 as the criterion for successful structure prediction, then DESTINI folds 478 (76%) targets versus 416 (66%) by TASSER. Similarly, these numbers are 361 (57%) and 321 (51%) at a TM-score > 0.6 for DESTINI and TASSER, respectively. Only at a very high TM-score cutoff of 0.9 does DESTINI have slightly fewer, 43, proteins compared to the 54 from TASSER. However, at this level, the difference in the TM-score is less relevant because all these models have very high quality; they typically have a less than a 2 Å RMSD from the native structure. The mean TM-score for the complete set is 0.601 by DESTINI, versus 0.569 by TASSER. A total of 171 (27%) targets have improved their TM-score by more than 0.1. These targets have a mean precision for the top *L* medium/long range contact predictions of 74.5% (Fig. 7B). Conversely, there are very few 36 (5.6%) targets with no improvement and a low TM-score < 0.4. They have a relatively low mean precision of 44.0% in contact prediction. The reason for these low-quality models is due to a combination of inaccurate template identification and mediocre contact prediction. Overall, it is clear that DESTINI is capable of improving model refinement even when a good structural template is already available.

### Comparison to other contact prediction methods

Finally, we compared the performance of DESTINI to several representative contact prediction methods. The benchmark set is composed of 66 domains from 50 targets evaluated during CASP12^35^. We only consider cases whose structural data were publicly available in the Protein Data Bank (PDB) at the time of this benchmark test. It is important to note that we removed from our training set all entries released after May 1^st^, 2016, the starting date of CASP12, and re-trained the network models with the reduced training set and a sequence library dated Feb 2016 for deriving the input features. These models are employed in the final benchmark tests. This procedure emulates the environment of CASP12. For each target, we made the prediction for the full sequence with no domain partitioning performed. Domain partitioning was only performed for evaluation using the boundary provided by the assessors.

**Figure 8:**
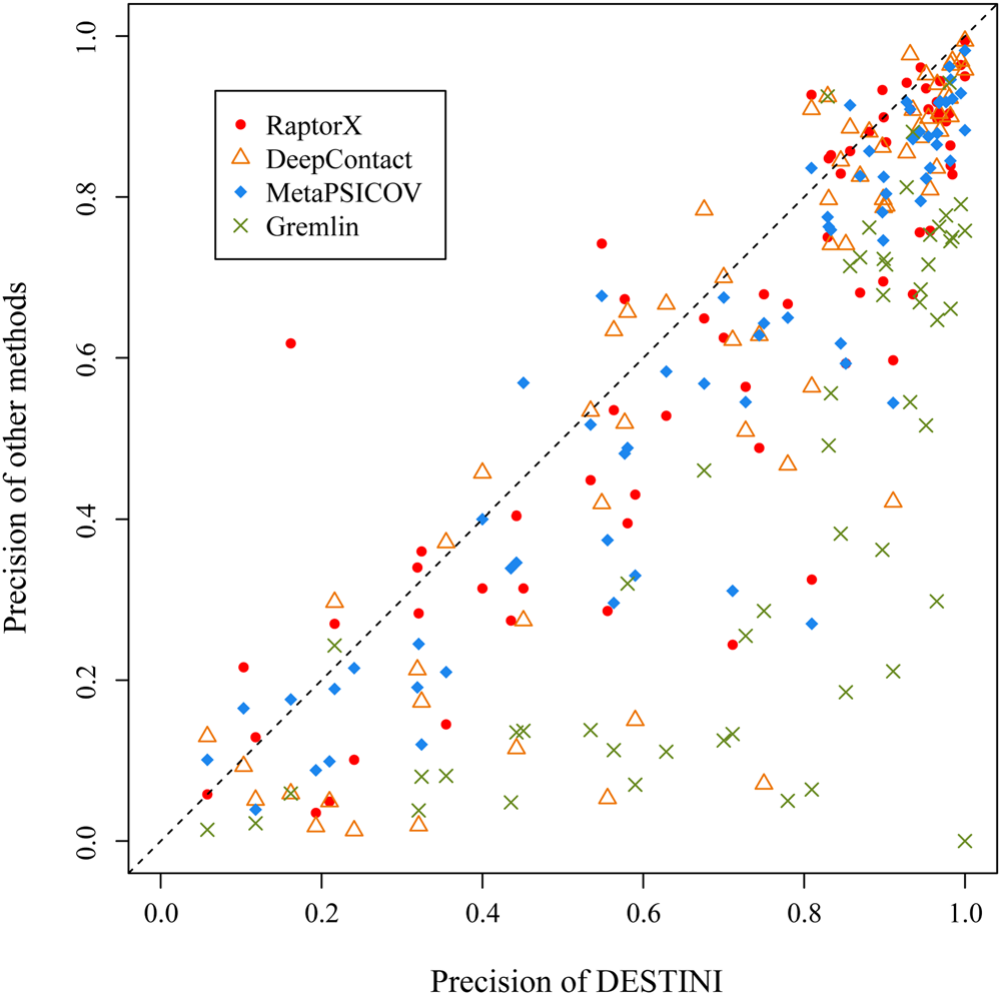
DESTINI *vs* four representative contact prediction methods on 66 CASP12 target domains. The comparison is based on the precision of the top *L*/2 predicted contacts provided by each method. Note that some methods, notably Gremlin, provide predictions for only a subset of targets. These missing cases are ignored for these methods.

The benchmark results are shown in Table 1 and Fig. 8. Overall, DESTINI significantly outperforms the other methods. For the top *L*/2 medium or long-range contacts, the mean precision of DESTINI is 70.1% versus 62.3% of RaptorX, the top ranked method in CASP12, 61.3% of DeepContact, which also employs a deep-learning algorithm, 60.7% of MetaPSICOV, the contact prediction leader in CASP11, and 42.8% of Gremlin, a standalone co-evolutionary analysis method. For the top *L*/5 medium or long-range predictions, the mean precision is 78.8% for DESTINI, compared to 69.6%, 68.1%, 69.3%, and 47.1% for RaptorX, DeepContact, MetaPSICOV, and Gremlin. With the exception of very few targets, Fig. 8 demonstrates that DESTINI performs better for the vast majority of targets compared to other methods. Overall, the benchmark test suggests that the performance of DESTINI is among the best, if not the best, contact prediction method.

**Table 1:** Mean precision of different contact prediction methods on CASP12 targets.

Method	# of Targets	Long*		Medium/Long	
		*L/5*	*L/2*	*L/5*	*L/2*
DeepContact	65	0.625	0.547	0.681	0.613
Gremlin	48	0.439	0.395	0.471	0.428
MetaPSICOV	66	0.599	0.511	0.693	0.607
RaptorX Contact	66	0.601	0.526	0.696	0.623
DESTINI	66	0.680	0.597	0.788	0.701

*Long (Medium/Long) denote long (medium or long) range contact predictions. The number in bold marks the best performance in each category (column).

## Discussion

Previously, it has been suggested that virtually all single-domain protein folds have been solved and deposited in the PDB^40,42^. Ideally, if one can correctly identify the corresponding template for a given target, one essentially solves the structure prediction problem for single-domain proteins. In practice, however, for some protein targets, it is surprisingly difficult to detect their structural homologs or analogs with a threading-based algorithm, when their relatively close homologs are not available. The reason is that certain similar but not identical contact patterns appearing in many folds are incorrectly aligned by threading algorithms, resulting in incorrect structural predictions. Thus, one expects that more accurate contact prediction could alleviate this issue. Indeed, with the more accurate contact prediction achieved through deep-learning, DESTINI clearly demonstrates that these contact predictions result in computational models with significantly better quality. In a large benchmark test on 606 “glass-ceiling” targets, only considering the top model, DESTINI is capable of predicting native-like folds for 37% of targets, compared to only 9% of targets by TASSER. Among these targets, the mean TM-score is 0.539, indicating a highly likely correct fold, versus 0.456 by TASSER. Moreover, even for “easy” targets whose correct template is most likely revealed by threading algorithms, DESTINI can further refine their models with its more accurate contact predictions. In a set of 636 easy targets, DESTINI generates native-like structural models for 89% of targets versus 80% by TASSER. If one uses a higher TM-score > 0.5 as the cutoff, then DESTINI folded 478 (76%) targets versus 416 (66%) by TASSER. Overall, it is clear that the incorporation of a deep-learning algorithm into protein structure prediction significantly elevates the accuracy of computationally derived structural models.

How does deep-learning improve the accuracy of contact prediction? A tertiary fold is determined by the packing of secondary structural segments, usually displaying patterns as clusters on a contact map. It appears that co-evolutionary coupling analysis is capable of discovering some elements of most native clusters, when there are enough diverse sequences available for analysis. A deep-learning approach such as DESTINI can further dramatically boost the accuracy of contact predictions by identifying more contacts within these clusters and eliminating spurious isolated contacts. In about half of the cases of the “glass-ceiling” set, DESTINI detects the same set of clusters as CCMPred, and only in about 30% of cases, did DESTINI identify new clusters not found by CCMPred. However, on average, DESTINI predicts more than twice the number of contacts per cluster than CCMPred. Moreover, it is interesting to see that most false positives (71%) by DESTINI surround native clusters, rather than being far away from these native contact clusters as was generated by CCMPred. This also helps to reduce egregious side-chain contact errors for use in modelling, as the predictions by DESTINI are more consistent and less noisy. Taken together, it appears that the deep-learning algorithm generates coherent, native-like contact patterns.

In real-world applications, one persistent question is how many medium/long range contacts are required in order to predict a correct fold? For a single-domain protein, our benchmark results suggest that a contact prediction depth *D* higher than 0.2 (that is *L*/5 contacts) for confident medium/long-range predictions is a good indicator for a successful structural prediction. In our test, the success rate is 69% at this criterion. Of course, this does not apply to multiple-domain proteins, which may or may not require very long-range contacts depending on the mutual arrangement of the domains. In addition, it should be noted that these statistics were based on our folding procedure, and other modelling approaches may see different performance even for the same contact predictions, depending on how a given modelling method uses the contact information.

Two deep-learning neural network models, RaptorX-Contact^33^ and DeepContact^34^, have been proposed. RaptorX-contact employs around 30 convolutional residual blocks like our approach here, whereas DeepContact employs 9 fully convolutional layers but lacks the residual circuit design. In the blind test CASP12, both approaches achieved good performance, and ranked in the first and third place, respectively^35^. Nevertheless, our approach exceeded their performance in a benchmark test emulating the CASP12 environment. One main difference is that we do not have a dedicated 1D convolutional component that is present in both RaptorX and DeepContact. In our tests, this 1D convolutional component may benefit when the network is relatively “shallow” with few 2D convolutional layers, but the advantage disappears when deep 2D layers are added in. In addition, it appears that the accuracy on the validation set is saturated when the number of 2D layers reaches a certain level. Indeed, we found virtually no improvement when we increased the neural network model from 40 2D convolutional layers to 60 2D layers. Thus, our model represents a succinct deep-learning design with good performance.

The co-evolutionary coupling signal is the most important contributor to the deep-learning models. Using this feature alone, it yields about 82% of the precision achieved when employing the full feature sets. Another interesting observation is that deep-learning dramatically improves the predictions made by using the raw co-evolutionary scores alone. Moreover, it appears that insights gained by statistical co-evolutionary analysis could not be trivially replaced by providing the raw frequency distributions from multiple sequence alignments, which CCMPred used for deriving the coupling scores. We attempted to use raw frequency scores to improve deep learning-based contact prediction but did not obtain meaningful improvement. What deep learning does, is to increase the coverage and coherence of predicted contacts between secondary structure elements and removes spurious isolated contacts relative to CCMPred. Thus, we not only have a better contact prediction approach but have elucidated how deep learning works to improve the quality of the resulting predicted contacts.

## Methods

### Deep-learning network

The problem of protein residue-residue contact prediction is essentially a pixel level classification on a 2D image, but it has many more channels (or features, see below) than a color image. To solve this problem, we applied ideas from deep-learning algorithms based on artificial neural networks^31^ in DESTINI. We employ a fully convolutional residual neural network consisting of 40 convolutional layers (Fig. 1). After initial processing, the input 2D features are fed to a single 2D convolutional and a rectifier activation layer, followed by 19 identical residual blocks connected sequentially. Each residual block consists of two 2D convolutionary layers, two rectifier layers, and an addition layer. For each residual block, the inputs ***x***_***i***_ are transformed into ***F(x***_***i***_***)*** **+** ***x***_***i***_ prior to the second activation (see Fig. 1), where ***F(x***_***i***_***)*** is the residual function^31^. If the new convolutional layers after ***x***_***i***_ reduce the training error, ***F(x***_***i***_***)*** should deliver a meaningful value; otherwise, then it is approximated by zero. Thus, residual blocks provide an effective way to train a deep neural network. In our implementation, each convolutional layer is composed of 64 filters with a kernel size of 3 × 3. Empirically, we found that this design gives rise the best results. Note that our network does not have any dropout layer, due to the fact that we need pixel-level resolution, unlike image segmentation problems^32^. Our design also does not contain any batch normalization layers, because the network takes an input protein sequence of arbitrary length. After 19 convolutionary blocks, the outputs are transposed and averaged prior to the last 2D convolutionary layer that outputs three channels, representing non-contact, contact, and ignored pairs. The latter is reserved for missing residues (gaps) in a protein’s structure. The layer of average operation guarantees that the predictions are symmetric and also helps to alleviate noise. Finally, a softmax layer is employed for calculating the probability scores^43^. For training, we employed the cross-entropy as the loss function. Our network model takes proteins of variable lengths. The DESTINI contact prediction is implemented with TensorFlow^44^. The full network has a total of 1.4 million parameters from 102 layers, including 40 2D convolutional layers.

### Deep-learning features

The input features to the DESTINI contact prediction consist of three 2D features and three 1D features. 2D features are for pairs of protein amino acids, whereas 1D features are for each amino acid of the sequence. The most important feature is the co-evolutionary score from CCMPred^19^, which is implemented in a fast GPU version, an advantage over other co-evolutionary analysis methods. The other two 2D features are mutual information^45^ and protein residue-residue statistical pair potentials^36^. In order to generate co-evolutionary scores and mutual information, HHblits is employed to perform the sequence alignment^46^. The sequence library from UNIPROT Feb, 2016^47^ is employed for HHblits. Standard parameters recommended by CCMPred were employed for running HHblits. The three 1D features are PSI-BLAST sequence profiling scores^38^, predicted secondary structure and solvent accessibility predictions for each residue. For secondary structure prediction, we implemented a 10 layer 1D fully convolutional residue network, similar to the one described above except its dimension is 1D instead of 2D. It takes the PSI-BLAST sequence profile and the target sequence as the input. The output is a 3-state prediction, alpha-helix, beta-sheet, and coil, at each residue position. For each benchmark test, we retrained this 1D model for secondary structure prediction using appropriately reduced training sets that removed homologs. The mean accuracy for secondary structure prediction is about 85% on the cross-validation sets, about 79% on the glass-ceiling set and 81% on the easy set in benchmarks. Solvent accessibility is calculated with SolvPred^25^. Finally, the 1D features are converted to 2D features by concatenating 1D features of two residues. The total input features have 51 channels, including 40 from sequence profiles, 6 from secondary structure, 2 from solvent accessibility, and 3 2D features. Thus, compared to image recognition, our problem has a much larger number of channels than the 3 channels of a typical image.

### Structural modeling

Depending on the difficulty of the targets, we employed different strategies for modeling easy and hard targets. An easy target is defined as having an SP3 threading Z-score ≥ 6.0^48^ whereas a hard target has a Z-score < 6.0. Regardless of target difficulty, the top 30 SP3 threading template models are used for starting structures in the TASSER simulation^49^. For easy targets, contacts are combined from the template models derived in the standard TASSER approach^49^ and the ones predicted by this deep learning method. Distance restraints derived from template models are kept unchanged. For hard targets, we utilize only the contacts predicted in this work and the threading templates are used only as starting structures. For each DESTINI predicted contact pair for hard targets, we also apply a distance restraint of 8.0 Å between the side chain centers of mass. Standard TASSER simulations were carried out for each target. Subsequently, simulation trajectories were clustered using the SPICKER approach^50^, and the cluster centroid of the largest cluster was used as the predicted top model of the target.

### Testing data sets

The “glass-ceiling” (hard) and easy data sets are collected from our previous study^14^. We removed entries marked as obsolete in the PDB from the original sets, and randomly selected proteins in the easy set so that the number of easy targets is approximately the same as the “glass-ceiling” set. A total of 1,237 proteins are selected, including 606 and 631 targets in the hard and easy sets, respectively. Both sets are composed of mainly single-domain proteins with lengths up to 200 residues. The CASP targets are retrieved from the CASP website (http://predictioncenter.org/). This set is composed of 50 targets.

### Training/validation data sets

The training/validation data sets are derived from the Feb 2017 release of PISCES, culled from the PDB at 25% sequence identity and better than 2.0 Å resolution^51^. Only sequences with lengths between 30 and 700 residues are retained. To create the training data sets for side chain contact prediction testing purposes, we further remove homologs that share 30% or higher sequence identity from the PISCES library, and obtain 7,732 and 7,585 entries for benchmarking DESTINI’s performance on the hard and easy sets, respectively. For CASP12 targets, all entries released after May 1st, 2016 are removed from the original library, resulting in a training set of 7,638 entries.

In each training session, the retained entries are subsequently split into two subsets, 800 cases as the validation subset, and the remaining as the training subset. The validation cases are used primarily to prevent overfitting and to select appropriate hyper-parameters, including the learning rate, momentum, and the weight decay parameter for the L2 regularization. We did not perform extensive brutal-force search for the best hyper-parameters, but rather selected a set of reasonably good parameters from hundreds of training runs. For each benchmark test, the training/validation subset split described above was conducted 5 times under the condition that entries from any two validation subsets have no intersection. Since the number of non-local contacts is very small, on average, about 1.5% of all possible non-local pairs, the contact classification problem is intrinsically unbalanced. While one can apply a higher weight on true contacts, this usually leads to higher recall but little improvement in the overall ranking for true contacts. The latter is more relevant to real applications. In our benchmark tests, we used a weight of 1 and 4 on each of five training/validation subsets, which yields a total of 10 models in each benchmark test. The final probability score of contact prediction is the arithmetic mean of these 10 models. This consensus approach slightly improves the prediction accuracy by a couple of percentage points.

### Contact pattern analysis

Contact patterns are obtained by an automated, unsupervised clustering procedure performed with HDBScan^52^. This is a hierarchical, density-based clustering algorithm that can be applied to a large dataset with noise. The idea of the algorithm is described online at https://hdbscan.readthedocs.io/en/latest/how_hdbscan_works.html. In our application, a minimal number of 3 points is required for each cluster. Cluster coverage is defined as the number of predicted clusters divided by the total number of clusters observed in the native structure. Contact coverage is defined by the number of predicted contacts within the clusters divided by all native contacts observed within the clusters.

## Data Availability

Benchmark data sets and a DESTINI webserver are available at http://pwp.gatech.edu/cssb/destini.
